# Zika structural genes determine the virulence of African and Asian lineages

**DOI:** 10.1080/22221751.2020.1753583

**Published:** 2020-05-22

**Authors:** Bruno T. D. Nunes, Camila R. Fontes-Garfias, Chao Shan, Antonio E. Muruato, Jannyce G. C. Nunes, Rommel M. R. Burbano, Pedro F. C. Vasconcelos, Pei-Yong Shi, Daniele B. A. Medeiros

**Affiliations:** a Department of Arbovirology and Hemorrhagic Fevers, Evandro Chagas Institute, Ministry of Health, Ananindeua, Brazil; b Department of Biochemistry & Molecular Biology, Galveston, TX, USA; c Department of Microbiology & Immunology, Galveston, TX, USA; d Institute for Human Infections & Immunity, Galveston, TX, USA; e Institute for Translational Science, Galveston, TX, USA; f Sealy Institute of Vaccine Sciences, Galveston, TX, USA; g Sealy Center for Structural Biology & Molecular Biophysics, Texas Medical Branch, Galveston, TX, USA; h Department of Pathology, Pará State University Belém, Brazil; i Post Graduation Program in Virology, Evandro Chagas Institute Ministry of Health, Ananindeua, Brazil; j Health Sciences Institute, Belem, Brazil; k Biological Sciences Institute - ICS, Federal University of Pará, Belem, Brazil

**Keywords:** Zika, virulence, lineages, African, Asian

## Abstract

The Asian lineage of Zika virus (ZIKV) is responsible for the recent epidemics in the Americas and severe disease, whereas the African lineage of ZIKV has not been reported to cause epidemics or severe disease. We constructed a cDNA infectious clone (IC) of an African ZIKV strain, which, together with our previously developed Asian ZIKV strain IC, allowed us to engineer chimeric viruses by swapping the structural and non-structural genes between the two lineages. Recombinant parental and chimeric viruses were analyzed in A129 and newborn CD1 mouse models. In the A129 mice, the African strain developed higher viremia, organ viral loading, and mortality rate. In CD1 mice, the African strain exhibited a higher neurovirulence than the Asian strain. A chimeric virus containing the structural genes from the African strain is more virulent than the Asian strain, whereas a chimeric virus containing the non-structural genes from the African strain exhibited a virulence comparable to the Asian strain. These results suggest that (i) African strain is more virulent than Asian strain and (ii) viral structural genes primarily determine the virulence difference between the two lineages in mouse models. Other factors may contribute to the discrepancy between the mouse and epidemic results.

## Introduction

Zika virus (ZIKV) is an enveloped virus belonging to the genus Flavivirus of the Flaviviridae family that has reemerged as a human pathogen with epidemic potential and severe neurological disorders such as Guillain-Barré syndrome and Zika congenital syndrome (microcephaly and others congenital defects) [1,2]. ZIKV has a positive single-strand RNA genome of about 11,000 nucleotides encoding three structural proteins (capsid [C], pre-membrane/membrane [prM/M], and envelope [E]) and seven nonstructural proteins (NS1, NS2A, NS2B, NS3, NS4A, NS4B, and NS5). The structural proteins form viral particles. The non-structural proteins participate in viral replication, virion assembly, and evasion of the host immune response [3]. Besides ZIKV, many other flaviviruses are also significant human pathogens, including the four serotypes of dengue (DENV), yellow fever (YFV), West Nile (WNV), Japanese encephalitis virus (JEV), and tick-borne encephalitis (TBEV) viruses.

ZIKV is classified into African and Asian lineages, both related to one serotype. The Asian lineage has been responsible for the recent epidemics in the Americas and associated with severe disease. ZIKV spread began in Yap Island, Micronesia in 2007 when an epidemic was reported [4,5]. The virus moved to French Polynesia and other islands of the South Pacific regions and caused large epidemics in 2013–14. In 2015, ZIKV reached the Americas and caused millions of human infections [6]. In contrast, the African lineage has not been associated with epidemics or severe disease manifestations in humans. This has raised the question of whether Asian strains were more virulent than the African strains. If so, the differences in epidemic potential and pathogenesis might be caused by genetic variance. Indeed, acquisition of adaptive mutations has been reported to alter flavivirus tropism, virulence, and vector competence [7–9].

Experimental systems, including a reverse genetic system of ZIKV, animal models, and mosquito transmission models, have been developed to address these questions. For mouse models, A129 (lacking type 1 interferon α/β receptors) mice were reported to be susceptible to ZIKV infection and to develop neurological disease [10–12]. When pre-treated with antibodies against type-I interferon receptors, immunocompetent mice could also be infected by ZIKV [10]. In addition, human STAT2 knock-in mice (hSTAT2 KI) have been developed to support mouse-adapted ZIKV replication [13]. These experimental systems have allowed us to compare the pathogenic difference between the Asian and African lineages as well as to map the genetic determinants for the pathogenic difference. In this study, we report the construction of a full-length cDNA clone of an African ZIKV strain. This cDNA clone, together with our previously developed ZIKV Asian lineage infectious clone [14], allowed us to engineer chimeric viruses between the two lineages to investigate viral pathogenesis.

## Materials and methods

### Cells, viruses, and antibodies

BHK-21 and Vero cells were purchased from the American Type Culture Collection (ATCC; Bethesda, MD) and maintained in high-glucose Dulbecco’s modified Eagle’s medium (DMEM) supplemented with 10% fetal bovine serum (FBS) (HyClone Laboratories, South Logan, UT) and 1% penicillin/streptomycin at 37°C with 5% CO_2_. *A. albopictus* C6/36 and U4.4 cells were grown in RPMI1640 (Invitrogen) medium containing 10% FBS, 1% penicillin/streptomycin, 1% non-essential amino acids, and 1% tryptone phosphate broth at 28°C with 5% CO_2_. The parental ZIKV African strain DAK AR 41525 (GenBank number KU955591) was obtained from the World Reference Center for Emerging Viruses and Arboviruses at the University of Texas Medical Branch (Galveston, TX, USA) and was isolated in AP61 cells in 1984 from *Ae. africanus* mosquitoes from Senegal and passaged once in C6/36 cells. FSS130125 (GenBank number KU955593.1) was a ZIKV human isolate obtained from Cambodia. the FSS virus used in our study was obtained from an infectious cDNA clone as previously reported [14]. There are 1193 nucleotide (NT) changes between ZIKV DKR and FSS strains spread across the genome. Most of these changes are in the non-structural region (*n* = 889), especially in the NS5 region, which concentrate more than 1/3 of nucleotides changes in the non-structural region (*n* = 318). In the other hand, on the structural region, we have 284 NT differences mainly clustered on the E region (*n* = 179). These NT changes encode a total of 23 amino acid differences located in the coding sequence of structural genes and 47 in nonstructural proteins. The following antibodies were used in this study: a mouse monoclonal antibody (mAb) 4G2 cross-reactive with flavivirus E protein (ATCC) and the goat anti-mouse IgG conjugated with Alexa Fluor 488 (Thermo Fisher Scientific).

### Construction of an infectious cDNA clone of ZIKV DAK AR 41525 strain

**Figure 1.**
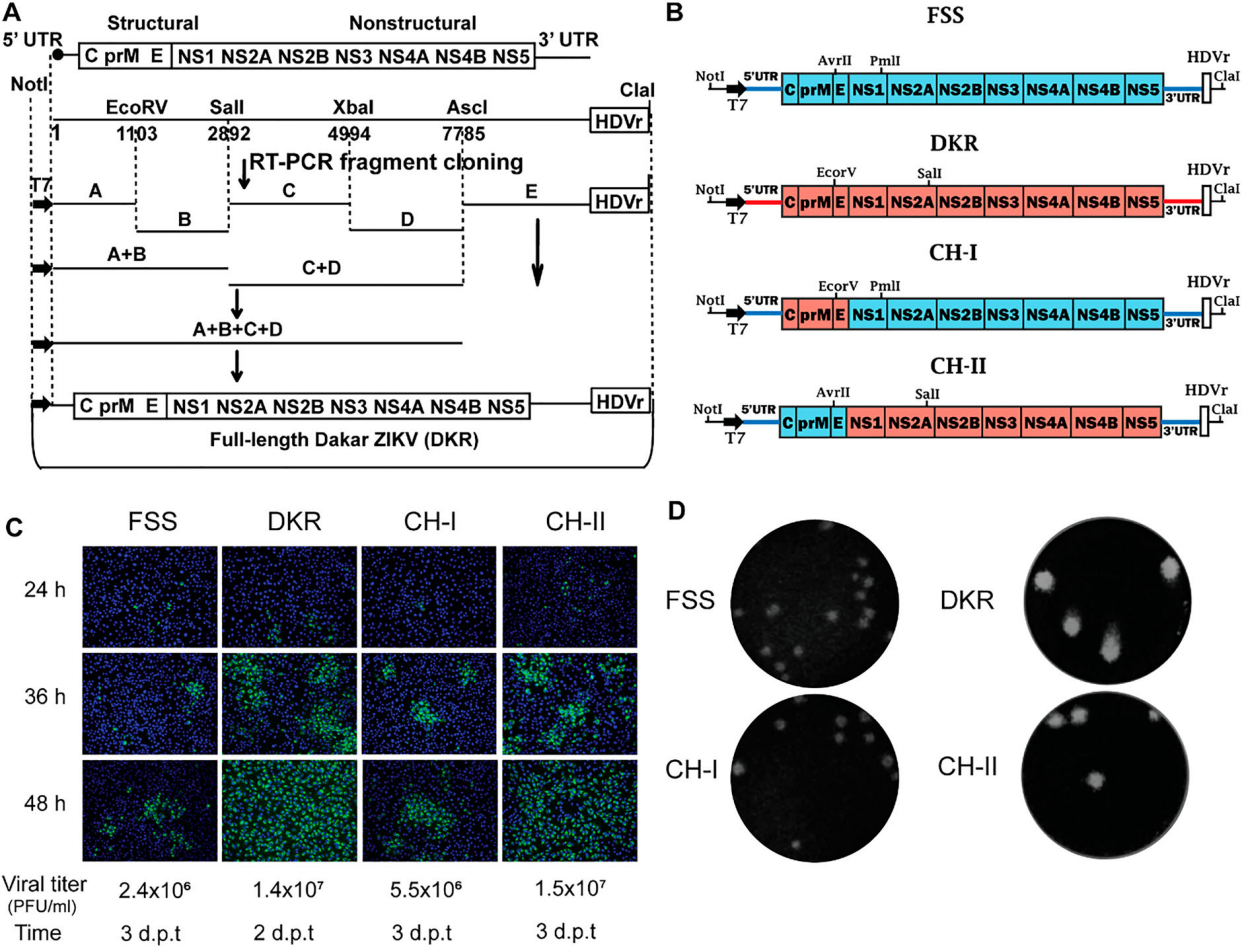
Construction of ZIKV DKR infectious clone and DKR/FSS chimeric viruses. (A) The strategy for constructing the full-length cDNA clone of ZIKV DKR. Genome organization, unique restriction sites, and their nucleotide positions are shown. Five cDNA fragments from A to E (represented by thick lines) were synthesized from genomic RNA using RT-PCR to cover the complete ZIKV DKR genome. Individual fragments were assembled to form the full-length cDNA clone of DKR (pFLZIKV-DKR). The complete DKR cDNA is positioned under the control of a T7 promoter for *in vitro* transcription. An HDVr sequence was engineered at the end of viral genome to generate an authentic 3’ end of viral RNA sequence. The numbers are the nucleotide positions based on the sequence of ZIKV African strain DAK AR D 41525 (GenBank ascension number KU955591). (B) Schematics of construction of ZIKV DKR and FSS strains chimeric viruses. Restriction enzyme sites used for cloning are indicated. The drawing is not to scale. (C) IFA of viral E protein expression in cells transfected with full-length ZIKV RNA and viral titres in culture fluids at day 2–3 Post-transfection. Vero cells were electroporated with 10 mg of genome-length DKR, FSS, CH-I, or CH-II viral RNAs. From 24 to 48 h p.t., IFA was performed to examine viral E protein expression using a mouse mAb (4G2). Green and blue represent E protein and nuclei (stained with DAPI), respectively. (D) Plaque morphologies of DKR, FSS, CH-I, and CH-II. Plaques were developed on a Vero cell monolayer after 4 days of infection.

Viral RNA from ZIKV African strain DAK AR 41525 (DKR) was extracted using QIAamp Viral RNA Kits (QIAGEN). The full genome of DKR virus was sequenced at the U.S. Army Medical Research Institute of Infectious Diseases and the University of Texas Medical Branch using Illumina NextSeq/MiSeq [15]. For the construction of an infectious cDNA clone of DKR virus, cDNA fragments covering the complete genome were synthesized from genomic RNA using SuperScript III (RT)-PCR (Invitrogen, Carlsbad, CA, USA). Figure 1A depicts the scheme to clone and assemble the full-length genome of ZIKV DKR strain. Plasmid pACYC177 (New England Biolabs, Ipswich, MA) was used to clone individual fragments A, B, C, D, and E as well as to assemble the full-length genomic cDNA. Bacterial strain Top 10 (Invitrogen) was used as the *E. coli* host for construction and propagation of cDNA clones. A standard cloning procedure was used, as previously reported for making ZIKV infectious clones [14]. The virus-specific sequence of each intermediate clone was validated by Sanger DNA sequencing before its use in subsequent cloning steps. The final plasmid containing full-length cDNA of ZIKV African strain (pFLZIKV-DKR) was completely sequenced to ensure there were no undesired mutations. Overlap PCR reactions were performed to engineer a T7 promoter and an HDVr (hepatitis delta virus ribozyme) sequence at the 5’ and 3’ ends of the complete viral cDNA to drive *in vitro* transcription and to generate the authentic 3’ end of viral sequence, respectively. All restriction endonucleases were purchased from New England Biolabs (Beverly, MA, USA).

### Construction of chimeric ZIKV cDNA clones

Two ZIKV infectious clones were used to construct the chimeras: pFLZIKV-DKR and pFLZIKV-FSS (Figure 1B). For construction of CH-I, two fragments (Fa and Fb) were initially amplified by PCR. Primers 14222F and 2505C-DKR/FSS were used to amplify the Fa fragment using pFLZIKV-DKR as a template. All primers are listed in Table 1. The Fa fragment contains a unique restriction enzyme site NotI followed by a gene cassette that includes the T7 promoter, ZIKV 5’UTR, the cDNA encoding the C, prM, and E genes from DKR strain. The Fb fragment was amplified using primer pair 2474V-DKR/FSS and 3498C using pFLZIKV-FSS as a template. Fb contains the first 1,009 nucleotides of NS1 coding region from the FSS13025 strain and a unique PmlI restriction enzyme site. Overlap PCR using primers 14222F and 3498C was performed to join the two fragments together, leading to Fab product that was later digested with NotI/PmlI and cloned into the pFLZIKV-FSS infectious clone. Finally, primers 5’UTR-42F and 5’UTR-92R were used to convert 5’UTR from DKR D41525 strain to FSS13025 strain by overlap PCR, resulting in the plasmid CH-I.

**Table 1.** Primers used in the construction of DKR infectious clones and ZIKV chimeric virus

Primer	Sequence
2476V-DK	CGGCTGTTTCTGCTGATGTTGGGTGCTCGGTGGAC
2502C-DK	CCTTTCCACGGCTGTTTCTGCTGATGTTGGGTGC
NotI-839V-DK	TCGCGGCCGCGATCAAGGTTGAAAATTGGATATTTAG
ClaI-3881C-DK	CGAATCGATAGCACGAAGCCAGGGCTAGCAG
3'UTR 10754F-DK	CCAGGCACAGATCGCCGAACAGCGGCGGCCGGTGTG
3'UTR 10789R-DK	CACACCGGCCGCCGCTGTTCGGCGATCTGTGCCTGG
5'UTR 46F-DK	GCGAGAGCTAACAACAGTATCAACAGGTTTAATTTG
5'UTR 92R-DK	TTTCCAAATCCAAATTAAACCTGTTGATACTG
ClaI-10740R-DK	CGAATCGATAGAAACTCATGGAGTCTCTGGTCTT
NotI-9854V	TCGCGGCCGCCTGCCGCCACCAAGATGAACTGATTG
CHII- EcoRVOUT F	CATCGAAAGAGCAGGTGACATCACATGGGAAAAAG
CHII- EcoRVOUT R	CTTTTTCCCATGTGATGTCACCTGCTCTTTCGATG
NotI-44V	TCGCGGCCGCAGCGAAAGCTAGCAACAGTATCAACAG
2474V-DAKAR/FSS	CACGGCTGTTTCTGCTGATGTGGGGTGCTCGGTGGAC
2505C-DAKAR/FSS	CCGAGCACCCCACATCAGCAGAAACAGCCGTGGAAAG
136F-DAKAR/FSS	GATTCCGGATTGTCAATATGCTAAAACGCGGAGTAG
167C-DAKAR/FSS	TCCGCGTTTTAGCATATTGACAATCCGGAATCCTCC
357F-DAKAR/FSS	TTCAAGAAAGACCTTGCTGCCATGTTGAGAATTATC
512C-DAKAR	GTACATGTAGTATGCACTCCCACGTCTAGTGATCTC
2476V-FSS/DAKAR	CAGCCGTCTCTGCTGATGTTGGGTGCTCGGTGGAC
2505C-FSS/DAKAR	GCACCCAACATCAGCAGAGACGGCTGTGGATAAG
ClaI-4434c-DAKAR	CGAATCGATTGTTTCCAGTGACTTCCGCGTC
NotI-4360V-DAKAR	TCGCGGCCGCGAAAGAGTGTGGACATGTACATC
ClaI-7824c-DAKAR	CGAATCGATAGTCCTCCTGTGGCCACTCCATCC
NotI-7750V-DAKAR	TCGCGGCCGCAAGTCAGGCATCACCGAAGTGTG
10376c-DAKAR	GGTGCTTACAGCACTCCAGGTGTGGACCCTTCCTC
10362V-FSS/DAKAR	ACACCTGGAGTGCTGTAAGCACCAATCTTAGTGTTGTC
NotI-9035V-DAKAR	TCGCGGCCGCATAGCTGTGTGTACAACATGATG
ClaI-9079C-DAKAR	CGAATCGATACATGTACCAGATTGCGCGGCTGC
14222F	GGGGTACCCAGATTTCGTGATGCTTGTCAG
3498C	GCCTTATCTCCATTCCATACCAACAACC

A similar approach was used to construct plasmid CH-II. First, a SalI restriction site at position 1,533 was knocked out from pFLZIKV-DKR infectious clone using overlap PCR. Next, two cDNA fragments, Fc and Fd, were amplified by PCR. The Fc fragment was obtained with primers 14222F and 2505C-FSS/DKR using pFLZIKV-FSS as a template. Thus, Fc contained a unique restriction enzyme site NotI followed the T7 promoter, ZIKV 5’UTR and C, prM, and E coding sequences from FSS13025 strain. Fd was amplified with primers 2474V-FSS/DKR and 3058C-DK using pFLZIKV-DKR as a template. This fragment contained the cDNA sequence of the first 570 nucleotides of DKR NS1 and a unique SalI restriction site. Fc and Fd fragments were fused together using an overlap PCR reaction with primers 14222F and 3058C-DK, resulting in Fcd product. The Fcd DNA was digested with NotI/SalI and cloned into the pFLZIKV-DKR infectious clone. Finally, the SalI restriction site at position 1533 was restored by overlap PCR, resulting in the plasmid CH-II.

### In vitro RNA transcription and transfection

Full-genome ZIKV-DKR, ZIKV-FSS, and chimeric viral RNAs were *in vitro* transcribed using a T7 mMessage mMachine kit (Ambion, Austin, TX) from cDNA plasmids pre-linearized by ClaI. The RNA was precipitated with lithium chloride, washed with 70% ethanol, re-suspended in RNase-free water, quantitated by spectrophotometry, and stored at −80°C in aliquots. The RNA transcripts (10 µg) were electroporated into Vero cells following a previously described protocol [14].

### Immunofluorescence assay (IFA)

IFA was performed according to a previously described protocol [16]. Briefly, Vero cells transfected with viral RNA were grown in an 8-well Lab-Tek chamber slide (Thermo Fisher Scientific, Waltham, MA). At 24, 32, and 48 h post-transfection (p.t.), the cells were fixed in 100% methanol at −20°C for 15 min. After 1 h incubation in a blocking buffer containing 1% FBS and 0.05% Tween-20 in PBS, the cells were treated with a mouse monoclonal antibody 4G2 for 1 h and washed three times with PBS (5 min for each wash). The cells were then incubated with Alexa Fluor® 488 goat anti-mouse IgG for 1 h in blocking buffer, after which the cells were washed three times with PBS. The cells were mounted in a mounting medium with DAPI (4’, 6-diamidino-2-phenylindole; Vector Laboratories, Inc.). Fluorescence images were observed under a fluorescence microscope equipped with a video documentation system (Olympus).

### Virus replication and plaque assay

U4.4 (8 × 10^5^ cells/well), C6/36 (8 × 10^5^ cells/well), BHK-21 (4 × 10^5^ cells/well), or Vero (4 × 10^5^ cells/well) cells were seeded into a 12-well plate one day prior to infection. At 24 h post-seeding, cells were infected with equal amounts of ZIKV-DKR, ZIKV-FSS, CH-I, or CH-II virus at a multiplicity of infection (MOI) of 0.01. Each infection was performed in triplicate. After incubation at 30°C (C6/36 and U4.4 cells) or 37°C (BHK-21 and Vero) for 1 h, virus inoculum was aspirated, and cells were washed extensively with PBS to remove unbound virus. Afterward, 1 ml of fresh medium was added to each well. From day 1–5 p.i., supernatants were collected daily and clarified by centrifugation prior to storage at −80°C. Virus titres were determined using a plaque assay on Vero cells as previously described [17].

### Virulence in A129 mice

All animal experiments were approved by the University of Texas Medical Branch IACUC. A129 mice were used to examine the virulence of ZIKV-DKR, ZIKV-FSS, and chimeric viruses as previously described [18]. In brief, 8-week-old A129 mice were infected in cohorts of 7 mice for each virus with 10^3^ PFU (dose used in our previous studies [18–20]) via the intraperitoneal route. Calcium- and magnesium-free Dulbecco’s phosphate-buffered saline (DPBS; Thermo, Fisher Scientific) was used to dilute the virus stocks to the desired concentration. DPBS injection was used as a mock infection. Mice were monitored daily. On days 2, 3, 4, and 5 p.i., mice were bled via the retro-orbital (RO) sinus route after being anesthetized. Sera were clarified post-collection by centrifugation at 6,000 x g for 5 min and were immediately stored at −80°C. Viremia was quantified by plaque assay on Vero cells. At days 6 and 9 p.i., mice were euthanized; blood was collected by cardiac puncture, and tissues (heart, lung, liver, spleen, kidney, muscle, brain, testis, and eye) were harvested. Organ titrations were performed using the plaque assay as described above. Briefly, 500 µl of DMEM with 2% FBS and penicillin/streptomycin along with a steel ball were placed in a 2-ml Eppendorf tube. The organ (whole or part) was placed in the tube. Tubes were weighed, and organ weight was determined by subtracting the tube weight. Tissues were homogenized in a Qiagen TissueLyser II shaking at 26 hz/sec for 5 min. The homogenate was clarified by centrifugation for 5 min at 12,000 rpm and titrated on Vero monolayer using plaque assay. The titre was then adjusted for volume and organ weight to report the organ loads as plaque forming units per gram (PFU/g).

### Neurovirulence in newborn CD1 mice

Newborn outbred CD1 mice were used to examine ZIKV neurovirulence as previously described [19]. Groups of 1-day-old CD1 mice (*n* = 7–10) were infected intracranially (I.C.) with ZIKV-DKR, ZIKV-FSS, CH-I, or CH-II with serial tenfold dilutions from 1,000 to 10 PFU. Mice were monitored daily for morbidity and mortality. All doses were chosen based in our previous studies [18–20].

### Data analysis

All data were analyzed with GraphPad Prism v7.02 software. Data were expressed as the mean ± s.e.m. and comparisons of groups performed using two-way ANOVA. For the CD1 mouse survival curve analysis, we used log-rank Matel-Cox test as well as logrank test for trend for all four viruses. In addition, median survival and hazard ratio (Mantel-Haenszel test) was calculated by comparing the survival curves of CH-I and CH-II. *P* value of <0.05 was considered statistically significant.

## Results

### Construction of an infectious cDNA clone of ZIKV African strain DAK AR 41525

We chose the African strain DAK AR 41525 (DKR) and the Asian strain FSS13025 (FSS) to examine the biological differences between the African and Asian lineages of ZIKV. DKR strain was isolated in 1984 from *A. Africanus* mosquitoes from Senegal with one round of passage on C6/36 cells [15]. FSS strain was isolated from a 3-year-old patient from Cambodia in 2010 [21]. We have previously developed an infectious cDNA clone for FSS strain [14]. To map the determinants of virulence and vector competence for FSS and DKR strains, we first constructed an infectious cDNA clone of DKR strain. The African strain used in our study (DKR) has only been passaged two times as opposed from the prototype African strain MR766, which has been passaged in suckling mouse brains up to 149 times [22]. As outlined in Figure 1A, five RT–PCR fragments (A–E) spanning the complete viral genome were individually cloned and assembled into the full-length cDNA of the viral genome, resulting in pFLZIKV-DKR. The low-copy number plasmid pACYC177 (15 copies per *E. coli* cell) was chosen as a vector to clone the individual fragments as well as to assemble the full-genome cDNA. A T7 promoter and an HDVr sequence were engineered at the 5’ and 3’ ends of the complete viral cDNA for *in vitro* transcription and for the generation of the authentic 3’ end of the RNA transcript, respectively. pFLZIKV-DKR was completely sequenced to ensure no mutations were present in the engineered viral cDNA. Transfection of Vero cells with DKR RNA transcript produced an increasing number of cells expressing viral E protein from 24 to 48 h post-transfection (h.p.t.) (Figure 1C). Culture supernatants of the transfected cells contained infectious DKR virus (Figure 1D) with a titre of 1.4 × 10^7^ PFU/ml at day 2 p.t. (Figure 1C). These results demonstrate that the DKR cDNA clone can produce infectious recombinant ZIKV.

### Construction and characterization of chimeric ZIKVs in cell culture

Using the infectious cDNA clones of DKR and FSS strains, we constructed two chimeric viruses (Figure 1B): Chimera I (CH-I) contained the three structural (C-prM-E) genes from DKR strain in the backbone of FSS strain; and Chimera II (CH-II) contained the seven DKR non-structural genes (NS1 to NS5) in the FSS backbone. After transfecting equal amounts of viral RNAs (10 µg) into Vero cells, the two chimeric RNAs as well as parental FSS and DKR RNAs generated viral E protein-expressing cells; more E-positive cells were observed in the DKR and CH-II RNA-transfected cells than those in the FSS and CH-I RNA-transfected cells (Figure 1C). In agreement with the IFA results, DKR and CH-II RNA produced viruses with bigger plaque sizes than the FSS and CH-I RNA did (Figure 1D). All four RNAs yielded infectious viruses with titres ranging from 2.4 × 10^6^–1.5 × 10^7^ PFU/ml at day 2 or 3 p.t. when the transfected cells started to show cytopathic effects (Figure 1C).

**Figure 2.**
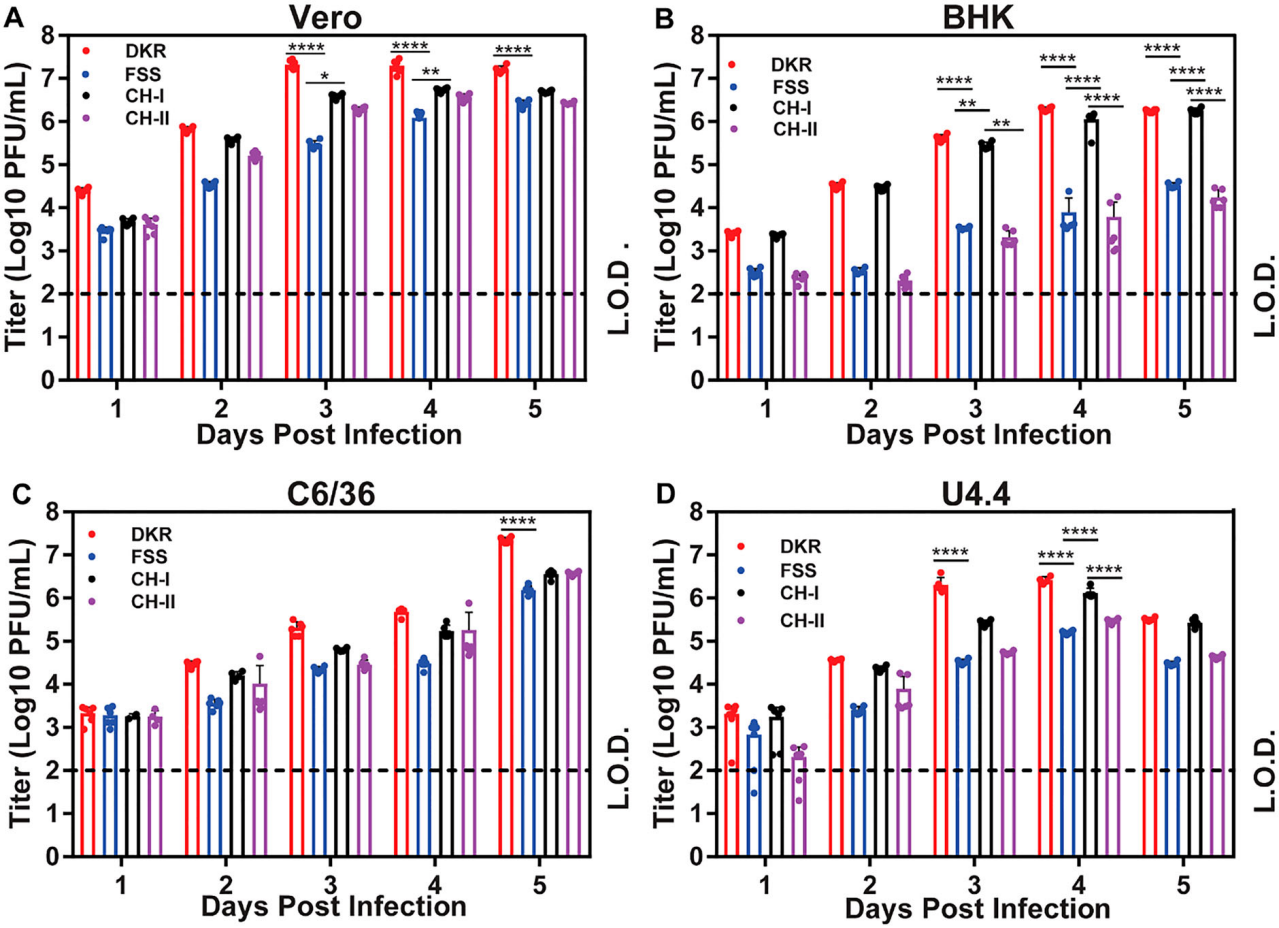
Characterization of DKR/FSS chimeric viruses in cell culture. Comparison of replication kinetics of DKR, FSS, CH-I, and CH-II in Vero (A), BHK-21 (B), C6/36 (C) and U4.4 (D) cells. All cells were infected at a MOI of 0.01. Viral titres were measured at indicated time points using plaque assays on Vero cells. Means and SDs from six independent replicates (indicated by dots) are shown. L.O.D., limitation of detection (100 PFU/ml). Two-way analysis of variance (ANOVA) was performed to analyze the statistical differences between the titres of DKR, FSS, CH-I, and CH-II at the indicated time points. *, *P* < 0.05 (significant); **, *P* < 0.01 (very significant); ****, *P* 0.0001 (extremely significant).

We compared the replication kinetics of FSS, DKR, CH-I, and CH-II on four different cell lines: African green monkey Vero, baby hamster kidney BHK-21, mosquito C6/36 (defective in RNAi pathway), and mosquito U4.4 (competent in RNAi pathway) cells. Between the two parental strains, DKR replicated to significantly higher titres than FSS did on all four cell lines (Figure 2). Overall, the chimeric viruses replicated at levels between the two parental strains, among which CH-I generated higher viral titres than the CH-II. These trends were more evident on Vero, BHK, and U4.4 cells than on C6/36 cells (Figure 2). Notably, between the two mosquito cell lines, the peak viral titre from the RNAi pathway-defective C6/36 cells (Figure 2C) was significantly higher than that from the RNAi pathway-competent U4.4 cells (Figure 2D), suggesting that RNAi pathway may play a role in suppressing viral replication in the U4.4 cells. Collectively, the results indicate that (i) DKR strain replicates more robustly than FSS strain and (ii) structural genes from DKR strain are much more efficient than the non-structural genes in enhancing the replication of FSS chimeric viruses in cell culture.

### Virulence in A129 mice

**Figure 3.**
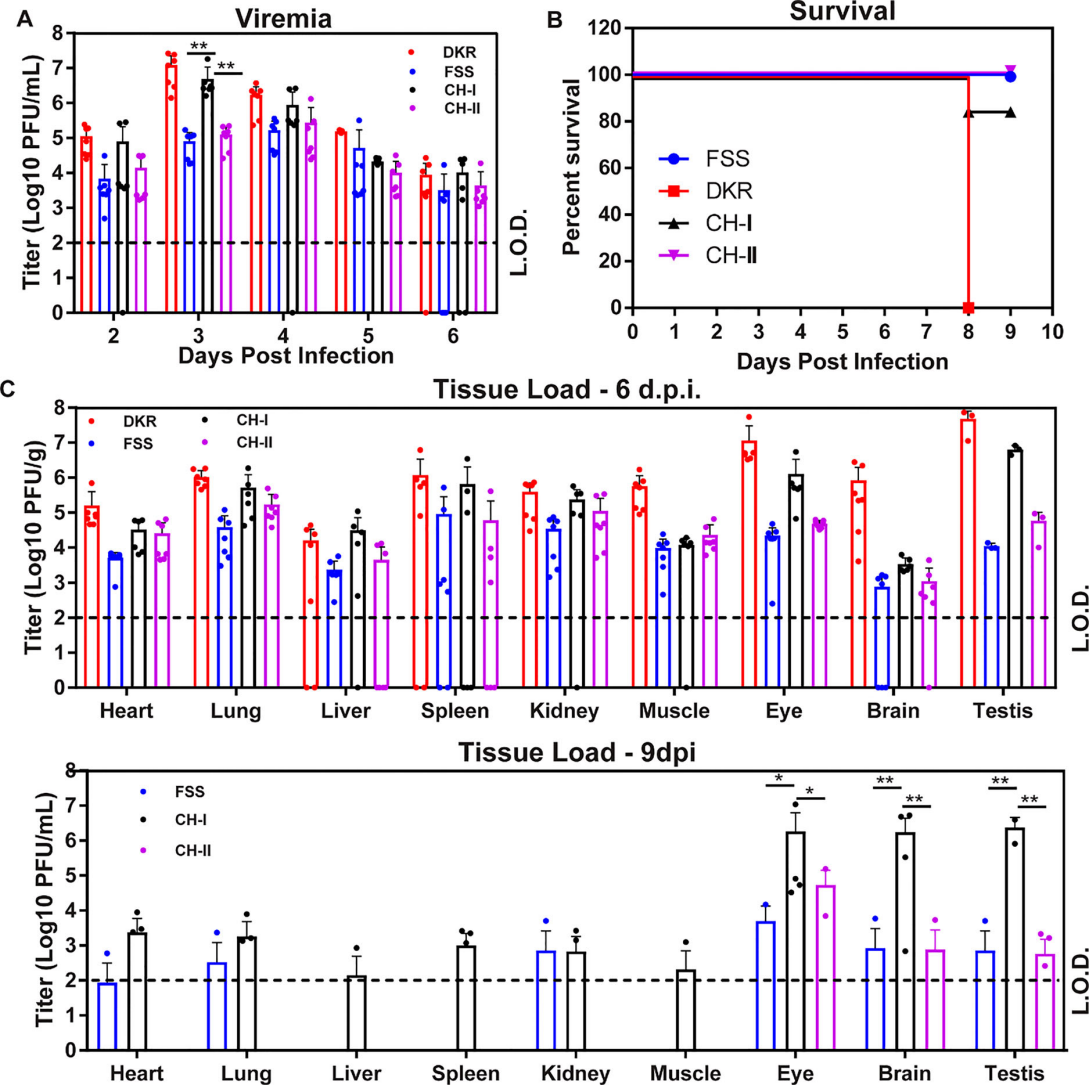
Virulence of DKR/FSS chimeric viruses in the A129 mice. Eight-week-old A129 mice (*n* = 7 mice per group) were intraperitoneally infected with 10^3^ PFU of DKR, FSS, CH-I, CH-II, or DPBS mock. (A) Viremia. The limit of detection (L.O.D.) for viremia was 100 PFU/ml. Each data point represents the mean level of viremia from 6 mice. (B) Survival analysis. Mice were euthanized once weight loss exceeded >20%. (C) Viral loads in organs of infected A129 mice. Organs were collected and homogenized on day 6 (top panel) and 9 (bottom panel) post-infection. The amounts of viruses were quantified on Vero cells using plaque assay. The averages results from four (at day 6 p.i.) or three (at day 9 p.i.) animals are presented. Bars denote standard error. All mice from group DKR died before collection date at day 9 post-infection. Two-way analysis of variance (ANOVA) was performed to analyze the statistical differences between the titres of each virus at the indicated time points and tissues. *, *P* < 0.05 (significant); **, *P* < 0.01 (very significant).

We examined the virulence of two parental and two chimeric viruses *in vivo* by using A129 mice, which lack interferon α/β receptors [11]. Equal amounts (10^3^ PFU) of individual viruses were inoculated into 8-week-old mice via the intraperitoneal (i.p.) route. The infected mice were tested for viremia (Figure 3A) and monitored for morbidity and mortality (Figure 3B). Mice infected with DKR and CH-I showed peak viremia of 1.2 × 10^7^ and 4.9 × 10^6^ PFU/mL, respectively, on day 3 post-infection. In contrast, FSS and CH-II showed significantly lower (*P *< 0.001) viremia of 8.1 × 10^4^ and 1.2 x10^5^, respectively, on day 3 p.i.; and peak viremia of 1.57 × 10^5^ and 2.1 × 10^5^ PFU/ml, respectively, at day 4 p.i. (Figure 3A). All animals from the DKR-infected group and 1/7 from the CH-I-infected group succumbed to infections, whereas no animal died from the FSS- and CH-II-infected groups (Figure 3B).

We also measured the viral burdens in various tissues and organs at days 6 and 9 after the A129 mice were infected intraperitoneally with 10^3^ PFU of DKR, FSS, CH-I, or CH-II. On day 6 p.i., all mice had high viral loads in every tested organ, with the highest titres in the testis, eye, spleen, and lung (Figure 3C, *top panel*). Among the four viruses, DKR and FSS consistently produced the highest and lowest organ viral loads, respectively. Although there was no statically significant difference in the titres of the two chimeric viruses in each tissue, CH-I mean titres in several organs was closer to DKR than to FSS, especially in the eye and testis. On day 9 p.i., only viral loads for FSS, CH-I, and CH-II were presented (Figure 3C, *bottom panel*) because all DKR-infected animals died on day 8 p.i. (Figure 3B). CH-I-infected mice exhibited detectable virus across all tissues at day 9 p.i., whereas FSS- and CH-II-infected animals showed lower levels of viral burdens at restricted organs, such as eye, brain, and testis (Figure 3C, *bottom panel*). Furthermore, CH-I titre in the brain increased almost 2-folds at day 9 compared to day 6 p.i.. The results suggest that the virulence of the four viruses are in the order of DKR > CH-I > CH-II ≈ FSS.

### Neurovirulence in newborn CD1 mice

**Figure 4.**
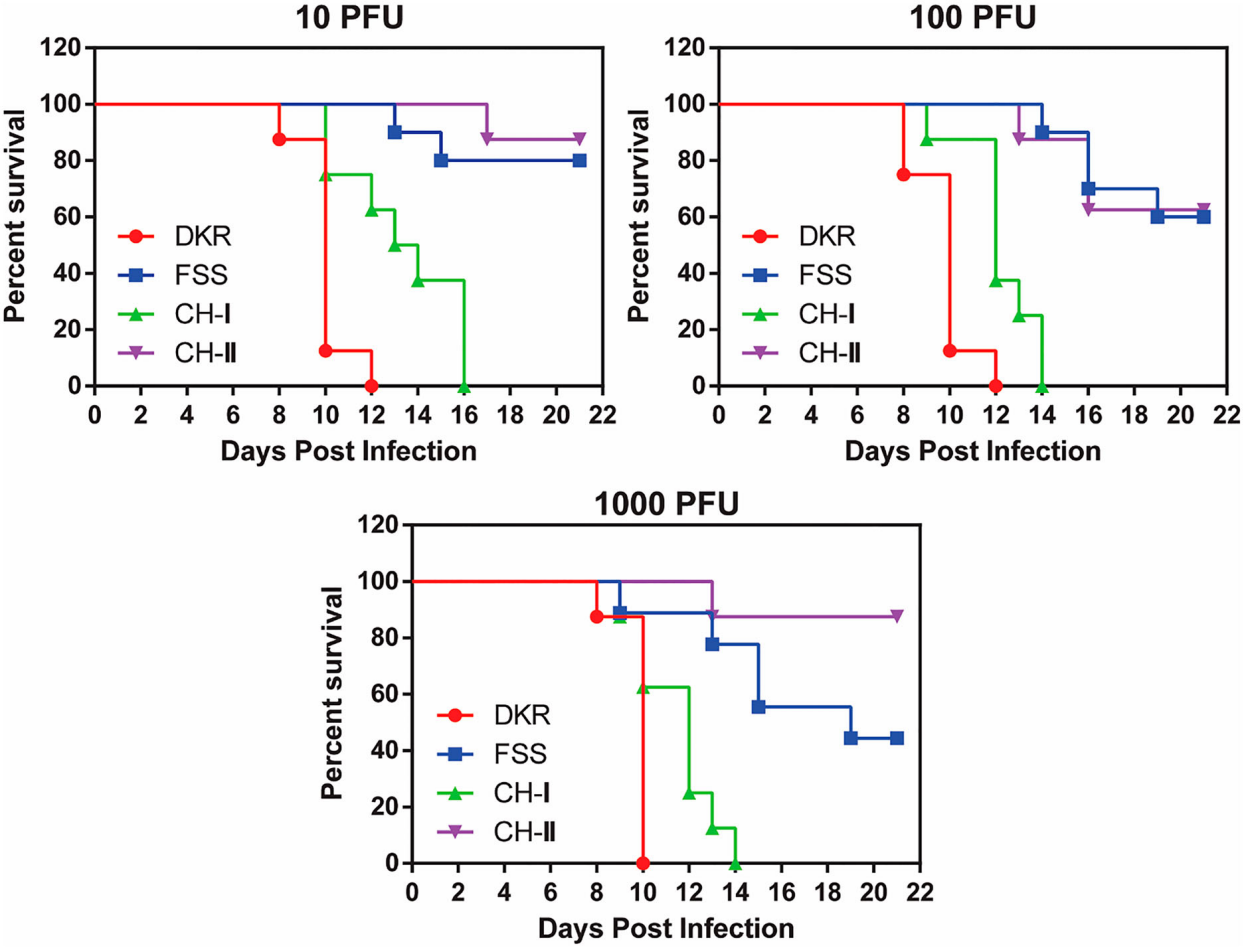
Neurovirulence of FSS, DKR, and chimeric viruses in newborn CD1 mice. Comparison of neurovirulence of DKR, FSS, CH-I, and CH-II viruses in one-day-old CD1 mice. Groups of newborn CD1 mice (*n* = 7–10) were injected via the I.C. route with 10–10^3^ PFU. For each virus, survival curves are presented for each infectious dose separately. Th survival curves were statistically different between the four viruses in all doses (*P *< 0.0001). The mean survival of CH-I at 10PFU dose was 13.5 days. Mouse infected with the smallest dose of CH-I had 21.29 times more risk of dying than those infected with the same dose of CH-II.

We examined the neurovirulence through intracranial (i.c.) infection of one-day-old CD1 mice. The newborn mice succumbed to each of the viral infections in a dose-responsive manner (Figure 4). When infecting at a dose range of 10–10^3^ PFU, DKR and CHI-I caused 100% mortality at days 10–12 and 14–16 p.i., respectively. In contrast, infections with CH-II and FSS at the same dose range resulted in 12.5–37.5% and 20–67% mortality, respectively (Figure 4). The results demonstrate the neurovirulence of the four viruses is in the order of DKR > CH-I > CH-II > FSS (*P *= 0.0031).

## Discussion

Since 2007, the world has witnessed an explosive epidemic associated with increased disease severity of ZIKV infection. However, the mechanisms behind the spread of ZIKV and its change in pathogenic profile remains to be understood. It has been suggested that Asian lineage of ZIKV may have evolved to generate higher and prolonged viremia in humans (leading to enhanced cross-placental infection and microcephaly) and/or enhanced mosquito transmission (leading to rapid virus spread and an increased number of human infections) [2,23]. This assumption is based on the fact that ZIKV African lineage has not been associated with the recent epidemics. Indeed, there is no epidemiological evidence that African lineage can cause developmental brain abnormalities in infants or severe disease manifestations. Several studies have provided explanations for the congenital malformations caused by ZIKV in South America, including changes in virulence after the virus left Africa. It is possible that genetic change have conferred an increased epidemic potential and pathogenesis in Asian lineage [6,24,25]. Our reverse genetic system has provided a tractable platform to define these genetic changes that are responsible for the increased pathogenesis and mosquito transmission.

ZIKV pathogenesis and its association with microcephaly remain poorly understood. Comparative studies have demonstrated that ZIKV strains of both lineages are able to infect different cell lines, primary cells [26,27], and brain organoids [28], and induce similar maternal, uterine, placental, fetal brain infection and fetal death in mice [29]. Interestingly, the African lineage strains have shown higher replication rate and cell death *in vitro* when compared to the Asian lineages strains [30–32]. In addition, the African strains are more pathogenic in non-pregnant mice [22,33–35], producing higher viremia and fatality in pregnant mice [10,36,37] and causing higher infection in the uterus and fetal organs in a porcine pregnancy model [38]. Moreover, the vertical transmission mouse model showed that infection with the African strain led to 100% fetal mortality, whereas abnormal fetuses from mice inoculated with the Asian strain was 53.2% [39]. The data indicate that even though both ZIKV lineages can induce congenital malformation, the African lineage seems to be more pathogenic and virulent than the Asian lineage in animal models, whereas the epidemiological data suggest the other way around.

We constructed a cDNA clone of the ZIKV African strain DAK AR 41525 to investigate the genetic markers that account for the biological difference between the two ZIKV lineages. The recombinant DKR virus also showed higher replication kinetics than FSS recombinant virus in both mammals and mosquito cells (Figure 2). In agreement with a previous study by Dowall et al. [33], DKR exhibited higher virulence than FSS in our mouse models, with increased viremia and organ viral loading in A129 mice and higher lethality in new-born CD1 mice (Figure 3). In addition, study of pathogenesis and host inflammatory immune responses in immunocompromised mice have shown that the African lineage causes exacerbated host inflammatory responses that result in increased tissue damage and faster cell death compared to the Asian lineage [35]. Together, these data confirm the higher virulence of African than Asian strains both *in vitro* and *in vivo*, as observed in other studies [10,28,30–32,38,40].

Compared with FSS and CH-II, DKR and CH-I produced higher viremia and organs viral loading in A129 mice. Consequently, FSS and CH-II infection did not kill any mice, whereas all mice infected with DKR and one mouse infected with CH-I died, the difference in the A129 mice survival curve between CH-I and CH-II, however, was not statistically significant (*P* = 0.3173) because the n was low due to A129 mice availability. DKR and CH-I also showed similar neurovirulence profile by killing all mice infected with all three doses, while some mice infected with FSS and CH-II survived. These results indicate higher virulence of the African over the Asian strains in animal models as previously reported [41] and suggest that the structural genes may be responsible for the virulence of African strains in these models. However, since only a single CH-I-infected A129 mouse died, while all the DKR-infected mice died, it is possible that nonstructural proteins may also contribute, to a less extent, to virulence. In addition, is important to note that we used the Asian backbone to construct the chimeras, which means both chimeras have the Asian FSS strain UTRs. ZIKV UTRs it is also important determinant of virulence, as the secondary genome structure could affect virus replication and contribute to the phenotypic differences observed between Asian and African lineages [42].

The Flavivirus structural proteins are primarily responsible for biding, entry, assembling and modulating viral infection cycle [3]. However, several studies have suggested that all three ZIKV structural proteins may also be involved in pathogenesis and neurovirulence. For instance, the prM have been related to increased infectivity of human and mouse neural progenitor cells (NPC) [9,43]. Indeed, our preliminary results obtained while further mapping the virulent determinants to individual structural genes suggest that prM seems critical to determine the virulence difference, thus prM might be a key determinant. However, more experiments are needed to further confirm this result. In addition, the E protein may responsible for enhanced virulence in mice and infectivity in mosquitoes [44] whereas the C protein is related to NPC apoptosis and cellular mRNA surveillance disruption mechanism that is required for normal brain size in mice [45,46].

Furthermore, comparing the data from 6 to 9 dpi, all mice infected by DKR died. CH-I could be detected in all organ, but FSS and CH-II could only be detected in the brain, eye, and testes on day 9 post-infection. The higher viremia and organs viral loading of DRK caused deaths up to 8 d.p.i., probably due to enhanced viral replication. On the other hand, CH-I developed higher organ viral loadings, whereas FSS and CH-II were in the process of viral clearance in various organs. We noticed there was no difference in organ tropism between DKR and FSS. Since the brain, eye, and testis are immunologically privileged, the immune response pattern in these organs is predominantly TH2 (cellular) type to avoid tissue damage [2,47]. Thus, ZIKV can efficiently replicate in these organs and prolong viral infection, as observed for the infection of CHI-I at day 9 post-infection.

Taken together, our results suggest that the African strain replicate at higher levels both *in vitro*, in cell lines derived from different host species, as well as *in vivo* in both IFN-α KO A129 mouse model and immunocompetent CD1 mouse model. In this sense, other studies have reported a similar pattern for African strains using porcine and non-human primate models [38,40,41]. However, even though some studies have shown that African lineage strains can also lead to higher infection rate and virus production in human neural [28,30,31] and placental trophoblast cells [32], the reason why African lineages seems to be less pathogenic in humans despite all the *in vitro* and *in vivo* data available remains to be understood. It is possible that other factors inherent to human populations may play a role.

In summary, our data reinforce that (i) there are intrinsic differences in the pathogenicity and virulence between the African and Asian ZIKV strains *in vitro* and *in vivo* and (ii) those differences may be related to sequence differences in the structural region of the genome. Additional studies are needed to pinpoint which sequence differences play a role in pathogenesis and their underlying mechanisms. The infectious cDNA clone of the African strain developed in this study will be useful for such studies.
